# SUMOylation of synaptic and synapse‐associated proteins: An update

**DOI:** 10.1111/jnc.15103

**Published:** 2020-07-05

**Authors:** Jeremy M. Henley, Richard Seager, Yasuko Nakamura, Karolina Talandyte, Jithin Nair, Kevin A. Wilkinson

**Affiliations:** ^1^ School of Biochemistry Centre for Synaptic Plasticity University of Bristol University Walk Bristol UK

**Keywords:** GTPases, ion channels, SUMOylation, synaptic plasticity, synaptic proteins

## Abstract

SUMOylation is a post‐translational modification that regulates protein signalling and complex formation by adjusting the conformation or protein–protein interactions of the substrate protein. There is a compelling and rapidly expanding body of evidence that, in addition to SUMOylation of nuclear proteins, SUMOylation of extranuclear proteins contributes to the control of neuronal development, neuronal stress responses and synaptic transmission and plasticity. In this brief review we provide an update of recent developments in the identification of synaptic and synapse‐associated SUMO target proteins and discuss the cell biological and functional implications of these discoveries.

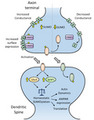

## Graphical abstract

In this Review, we highlight advances in the field of neuronal SUMOylation and discuss how SUMOylation regulates processes within pre‐ and post‐synapses. Enhanced SUMOylation of Na^+^ channels increases conductance and SUMO‐modified collapsin response mediator protein 2 stabilizes Na^+^ channel surface expression. DeSUMOylation of metabotropic glutamate receptors mGluR7 promotes internalization. At the post‐synapse, GluK2 activation leads to SUMOylation and internalization. mGluR activation leads to accumulation of Ubc9, but can also enhance SENP1 levels, likely leading to homeostatic SUMO modulation. Downstream effects include regulation of actin dynamics, increasing AMPA receptor expression and local translation. These findings provide new appreciation of the roles of SUMOylation in synaptic function and plasticity.

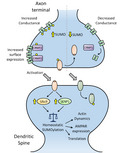

**Abbreviations**
AMPAR: α‐amino‐3‐hydroxy‐5‐methyl‐4‐isoxazolepropionic acid receptor
APP: amyloid precursor protein
BACE1: beta‐site amyloid precursor protein cleaving enzyme 1 (β‐secretase 1)
BDNF: brain‐derived neurotrophic factor
CAD: catecholamine A‐differentiated
CC2D1A: coiled‐coil and C2 domain‐containing 1A
CDK: cyclin‐dependent kinase
CPEB3: cytoplasmic polyadenylation element‐binding protein 3
CRMP2: collapsin response mediator protein 2
DAT: dopamine transporter
DRG: dorsal root ganglion
EPSC: excitatory post‐synaptic current
ERK: extracellular signal‐regulated kinase
FMRP: fragile X mental retardation protein
GlyR: glycine receptor
GPCR: G‐protein‐coupled receptor
HD: Huntington's disease
HEK293: human embryonic kidney 293
I_A_: transient‐inactivating A‐type potassium current
I_h_: hyperpolarization‐activated current
JNK3: c‐Jun N‐terminal kinase 3
KAR: kainate receptor
K_v_: voltage‐gated potassium channel
L‐AP4: L‐2‐amino‐4‐phosphonobutyric acid
LTD: long‐term depression
LTP: long‐term potentiation
M1 mAChR: M1 muscarinic acetylcholine receptor
MEK1: MAPK/ERK kinase 2
mEPSC: miniature excitatory post‐synaptic current
mGluR: metabotropic glutamate receptor
mHTT: polyQ‐expanded mutant Huntingtin
MLK3: mixed‐lineage protein kinase 3
nNOS: neuronal nitric oxide synthase
NO: nitric oxide
PD: Parkinson's disease
PIAS2: protein inhibitor of activated STAT 2
PKC: protein kinase C
PSD95: post‐synaptic density protein 95
Rhes: Ras homolog enriched in striatum
SAE1/2: SUMO‐activating enzyme 1/2
SENP: sentrin protease
SIM: SUMO‐interacting motif
SNARE: soluble NSF attachment protein receptor
SNI: spinal nerve injury
SUMO: small ubiquitin‐like modifier
TRPV1: transient receptor potential cation channel subfamily V member 1
Ubc9: ubiquitin‐conjugating protein 9

## 1 INTRODUCTION

SUMOylation is a highly dynamic post‐translational modification of lysine residues in target proteins. SUMOylation and deSUMOylation of specific proteins plays key roles in the signalling pathways involved in nearly all aspects of neuronal form and function (Henley, Craig, & Wilkinson, 2014; Schorova & Martin, 2016). SUMO modification of nuclear proteins has been extensively characterized (Jentsch & Psakhye, 2013), but multiple proteins in extranuclear compartments are also subject to SUMO modification, although in many cases a precise understanding of the mechanisms and consequences of these SUMOylation events is, as yet, less well established (Luo et al., 2013).

There have been a number of reviews and commentaries about SUMOylation of extranuclear proteins in neurons, most recently (Henley, Carmichael, & Wilkinson, 2018). However, this is a rapidly moving area of neuroscience and several new proteins that have direct relevance to synaptic function and dysfunction have recently been identified and/or validated as SUMO targets.

Here we focus predominantly on the SUMOylation of these recently identified synaptic and synapse‐associated proteins and also on targets that have not been extensively covered in previous reviews. This is not an exhaustive list, rather we have arranged the SUMO substrates into classes of proteins and discuss how these new findings add to our understanding of how SUMOylation can regulate a wide range of physiological and pathophysiological processes in neurons.

## 2 SUMO conjugation and deconjugation—a brief overview

In mammals there are three validated SUMO paralogues, SUMO1‐3. SUMOs are ~ 11kD proteins that are covalently conjugated to lysine residues in target proteins by an enzymatic cascade analogous to, but distinct from, the ubiquitin pathway (for extensive reviews of the SUMO pathway see (Flotho & Melchior, 2013; Hay, 2005; Wilkinson & Henley, 2010; Wilkinson, Nakamura, & Henley, 2010)). Briefly, SUMO2 and 3 are highly homologous, differing in only 3 amino acids, and are therefore collectively referred to as SUMO2/3, but SUMO2 and SUMO3 are only ∼48% identical to SUMO1. SUMO proteins are initially synthesized as inactive precursors that are first cleaved by a member of the SUMO protease family of proteins to expose a C‐terminal di‐glycine motif that is required for conjugation. SUMO is then activated for conjugation in an ATP‐dependent manner by the E1 enzyme, a dimer of SAE1/2, before loading onto Ubc9, the sole identified E2‐conjugating enzyme for SUMOylation. SUMO is then conjugated to the target lysine by Ubc9, a process that is facilitated in vivo by a number of identified E3 enzymes. Once conjugated, SUMO can be removed from target proteins by the actions of SUMO proteases, the most well characterized of which are the Sentrin/SUMO‐specific proteases (SENP1‐3, 5–7). Thus, the dynamic balance between SUMO conjugation, mediated by a restricted set of SUMOylation enzymes, and deSUMOylation mediated by SUMO proteases, controls substrate protein properties.

## 3 Expression and compartmentalization of SUMOylation and deSUMOylation enzymes in neurons

Neurons are morphologically complex, highly compartmentalized and exquisitely activity‐sensitive cells. The vast array of spatio‐temporally regulated events in neurons requires the sophisticated coordination of protein trafficking, retention and turnover. Given the wide acceptance of the importance of extranuclear protein SUMOylation, including at synapses, how SUMO enzymes are localized and regulated in these compartments is an important but still poorly understood field.

A recent paper used super resolution microscopy to assess the subcellular distributions of endogenous SUMO2/3 (and to a lesser extent SUMO1) and the SUMO‐conjugating enzyme Ubc9 in cultured neurons (Colnaghi et al., 2019). Consistent with all previous studies, they report that the majority of SUMO and SUMOylation enzymes are present in the nucleus, but SUMO1, SUMO2/3 and Ubc9 were also detected in both pre‐ and post‐synaptic compartments. More specifically, in primary hippocampal neurons, they showed that three separate antibodies raised against different regions of SUMO2/3 partially colocalized with the pre‐ and post‐synaptic markers synaptophysin and PSD95 respectively (Colnaghi et al., 2019). Moreover, they also specifically excluded the possibility that the presence of synaptic SUMO proteins was because of synaptically localized mitochondria, which contain a number of known SUMO targets (Henley et al., 2018). These data lend further support to key roles of SUMOylation in the direct regulation of synaptic activity.

**FIGURE 1:**
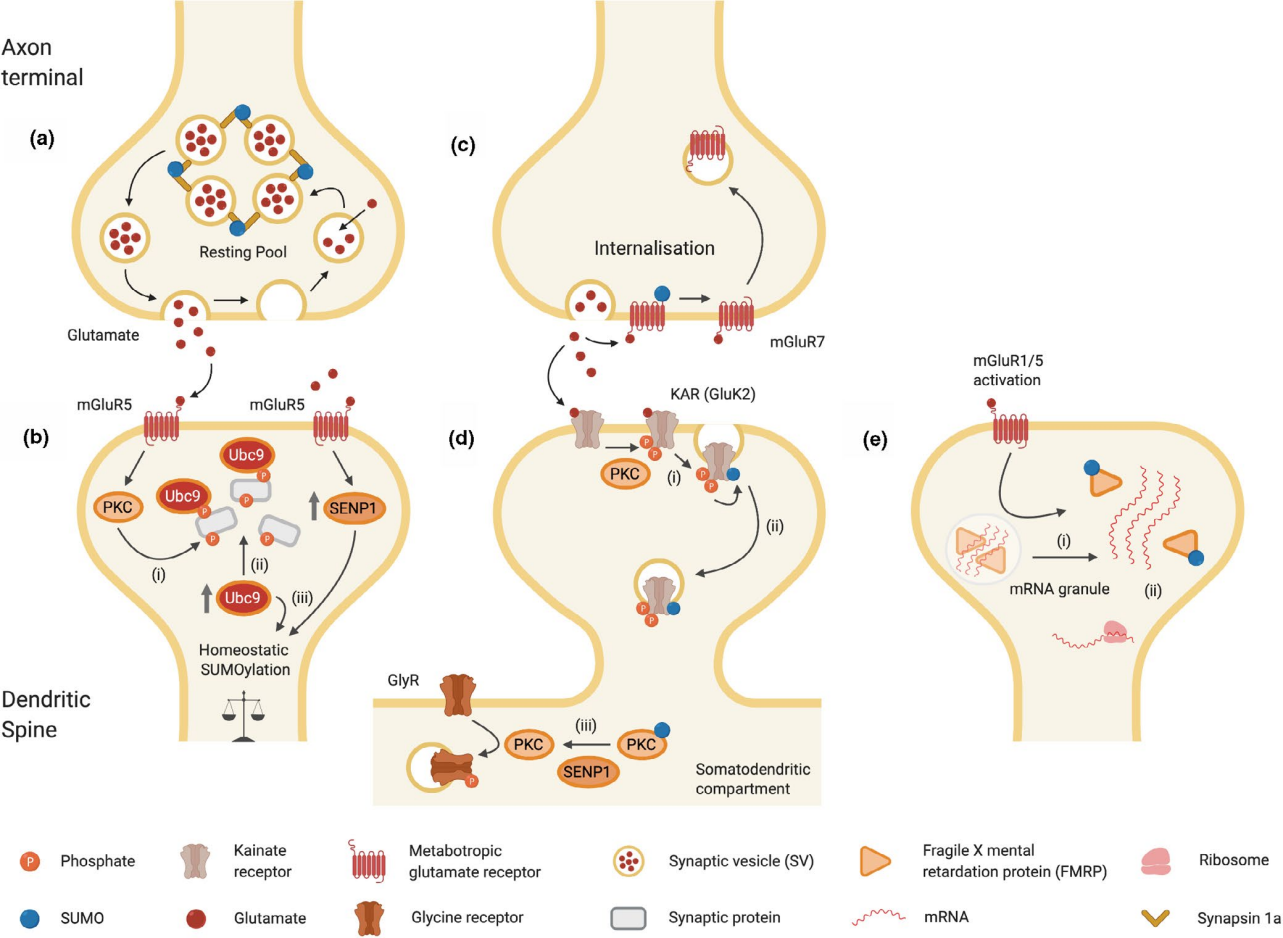
SUMOylation of synaptic receptor and receptor‐associated proteins. Schematic summarizing the functional effects of SUMOylation and deSUMOylation on selected synaptic proteins. (a) SUMOylation of the pre‐synaptic Synapsin 1a facilitates the re‐clustering/anchoring of synaptic vesicles after neurotransmitter release. (b) mGluR5‐dependent synaptic trapping of Ubc9 (i) Activity‐induced PKC activation phosphorylates synaptic proteins. (ii) Ubc9 binding to phospho‐proteins ‘traps’ Ubc9 in the synapse. (iii) Correspondingly, activation of mGluR5 increases dendritic SENP1, providing a homeostatic feedback mechanism. (c) SUMOylation of mGluR7 stabilizes its pre‐synaptic surface expression. Glutamate activation of mGluR7 results in deSUMOylation and internalization. (d) PKC phosphorylation of the Kainate receptor subunit GluK2 leads to SUMOylation (i) and internalization (ii). PKC SUMOylation inhibits its kinase activity whereas SENP1 deSUMOylation of PKC enhances activity leading to phosphorylation of glycine receptor (GlyR) and facilitates internalization (iii). (e) mGluR1/5 activation enhances SUMOylation of FMRP, facilitating dissociation from mRNA granules (i) and leading to increased local translation (ii)

It has also been reported that activation of the metabotropic glutamate receptor mGluR5, increases the synaptic residency time of the SUMO‐conjugating enzyme Ubc9 by transiently restricting Ubc9 diffusion out of the dendritic spine (Loriol et al., 2014). The mechanism underpinning this synaptic retention likely involves Ubc9 binding to PKC‐phosphorylated synaptic substrates resulting in diffusional trapping of Ubc9 and increased synaptic SUMOylation (Loriol et al., 2014). This activity‐dependent recruitment and retention of Ubc9 in spines may facilitate the dynamic up‐regulation of SUMOylation to modulate synaptic transmission and plasticity (**Figure** **1**).

The diffusion properties of the SUMO protease SENP1 in individual hippocampal spines has also been investigated, and it was recently reported that this too is regulated by mGluR5 activity (Schorova et al., 2019). Similar to Ubc9, mGluR5 activation reduced diffusion and caused an accumulation of SENP1 in dendritic spines (**Figure** **1**). The authors propose that the post‐synaptic increases in both Ubc9 and SENP1 act synergistically to homeostatically regulate synaptic protein SUMOylation (Schorova et al., 2019). Indeed, several SUMOylation enzymes, including SAE1, Ubc9 and SENP1, have been reported to undergo developmental and potassium chloride depolarization‐dependent redistribution in neurons (Loriol, Khayachi, Poupon, Gwizdek, & Martin, 2013). However, many of the other physiological and pathophysiologically relevant stimuli have not been investigated and exactly how neuronal activity leads to alterations in the localization of these enzymes, and the consequences for neuronal function, remain unclear.

Although primarily localized in the nucleus, SENP3 also has a widespread distribution in neurons including localization at mitochondria and synapses (Guo et al., 2013). Moreover, levels of individual SENPs are differentially regulated. For instance levels of SENP1 are increased whereas levels of SENP3 are reduced in neurons during ischemic cell stress (Cimarosti et al., 2012; Guo et al., 2013; Guo, Wilkinson, Evans, Rubin, & Henley, 2017), indicating both proteins are dynamically regulated and that there may be a reciprocal relationship between the activity and/or levels of SENP1 and SENP3. It is well established that both in vitro and in vivo models of ischemia lead to enhanced SUMOylation, particularly by SUMO2/3 (for review see (Bernstock et al., 2019)). Since SENP1 deconjugates both SUMO1 and SUMO2/3, and SENP3 targets SUMO2/3 (Hickey, Wilson, & Hochstrasser, 2012), how this reciprocal regulation leads to enhanced SUMO2/3 conjugation remains unclear, but it remains possible that they target different subsets of substrates, resulting in a net increase in SUMO2/3 conjugation driven by SENP3 loss (Guo et al., 2013), however this possibility needs experimental confirmation. Moreover, how ischaemia alternately regulates SENP levels and activity, and how this shapes the profile of modified SUMO targets to induce the neuronal response to stress, has not yet been extensively investigated.

### 3.1 SUMOylation of synaptic proteins

It was first reported that SUMOylation can regulate neurotransmitter release from isolated synaptosomes (Feligioni, Nishimune, & Henley, 2009) and subsequent studies have shown that SUMOylation controls proteins essential for pre‐synaptic neurotransmitter release, as well as post‐synaptic receptors, ion channels and proteins integral to synaptic function (Table 1; for review see (Henley et al., 2014)). For example synapsin 1a is a component of the pre‐synaptic SNARE machinery that modulates vesicle availability (Figure 1). Preventing synapsin 1a SUMOylation at Lys687 alters vesicle trafficking and exocytosis (Tang, Craig, & Henley, 2015). Furthermore, an autism and epilepsy‐associated mutation in synapsin 1a (Fassio et al., 2011) reduces its SUMOylation and displays similar functional defects to non‐SUMOylatable synapsin 1a, suggesting defective SUMOylation may lead to synaptic dysfunction in these disorders (Tang et al., 2015).

Although changes in the synaptic surface expression of α‐amino‐3‐hydroxy‐5‐methyl‐4‐isoxazolepropionic acid receptors (AMPARs) is a key aspect of plasticity in excitatory neurons, AMPAR subunits are not SUMOylated (Martin, Nishimune, Mellor, & Henley, 2007; Wilkinson, Nishimune, & Henley, 2008). However, theta burst stimulation, which induces long‐term potentiation (LTP) in cultured slices, leads to an increase in SUMO2/3 conjugation (Lee et al., 2014), and chemically inducing LTP with glycine stimulation in cultured neurons increases SUMO1 mRNA levels (Jaafari et al., 2013), indicating that plasticity induction can alter the protein SUMOylation pathway, in a manner dependent on the stimulation protocol and experimental system used. Furthermore, reducing SUMOylation by over‐expressing the catalytic domain of SENP1 or a dominant‐negative mutant of Ubc9 interferes with AMPAR trafficking, plasticity and hippocampal‐dependent learning (Jaafari et al., 2013; Lee et al., 2014) suggesting that proteins that mediate the activity‐dependent sorting of AMPARs are under the control of the SUMOylation pathway.

### 3.2 Synaptic neurotransmitter receptors and transporters

#### 3.2.1 G‐protein‐coupled receptors (GPCRs)

As set out in the examples cited below, there is increasing evidence that SUMOylation of G‐protein‐coupled receptors modulates downstream Ca^2+^ signalling and neurotransmitter release.

#### 3.2.2 mGluR7 and mGluR8

Metabotropic glutamate receptors (mGluRs) are class C, G‐protein‐coupled receptors that play a role in modulating synaptic transmission and neuronal excitability. They are divided into three groups and have been reviewed extensively (for example see (Niswender & Conn, 2010)). Group III mGluRs (mGluR4 and mGluR6‐8) are pre‐synaptic and suppress excitation. mGluR7 is an autoreceptor that inhibits glutamate release to modulate excitatory neurotransmission and synaptic plasticity (Enz, 2012; Mukherjee & Manahan‐Vaughan, 2013) (Figure 1). Several studies have reported an interaction between Group III mGluRs and SUMO pathway proteins, and demonstrated SUMOylation of their intracellular C‐termini in vitro (Dutting, Schroder‐Kress, Sticht, & Enz, 2011; Tang, El Far, Betz, & Scheschonka, 2005; Wilkinson et al., 2008). In clonal cell line expression systems mGluR8b is SUMOylated at Lys882 and Lys903, whereas mGluR7 is SUMOylated at Lys889 (Dutting et al., 2011; Wilkinson & Henley, 2011). More recently, SUMOylation of full‐length mGluR7 at Lys889 has been confirmed in brain and primary cortical neurons, and SUMOylation is decreased by the mGluR7 agonist L‐AP4 (Choi et al., 2016). Moreover, a non‐SUMOylatable mutant of mGluR7, or over‐expression of SENP1 in hippocampal neurons, caused enhanced mGluR7 internalization, suggesting that SUMOylation can stabilize surface expression of mGluR7 (Choi et al., 2016) (Figure 1).

#### 3.2.3 M1 muscarinic acetylcholine receptor (M1 mAChR)

Another GCPR proposed as a SUMO target is the M1 muscarinic acetylcholine receptor (M1 mAChR) (Xu et al., 2019) which plays important roles in learning and memory (Kruse et al., 2014). M1 mAChR has been reported to be SUMOylated by SUMO1 at K327, which is situated in the intracellular loop 3, and receptor activation with carbachol decreases this SUMO1‐ylation. However, it is notable that the SUMO conjugated M1 appears to resolve at the same molecular weight as non‐SUMOylated M1, which is difficult to explain. Nonetheless, these authors conclude that SUMO1‐ylation increases M1 mAChR ligand‐binding affinity, signalling and receptor endocytosis. Mutation of K327 to arginine attenuated M1 mAChR SUMOylation by SUMO1 and decreased ligand‐binding affinity and signal transduction. Molecular dynamics simulations suggest SUMOylation regulates mAChR by stabilizing the receptor in an active‐state conformation (Xu et al., 2019).

#### 3.2.4 Kainate receptors (KARs), Protein Kinase C (PKC) and Glycine receptors (GlyRs)

Kainate receptors are a subclass of ionotropic glutamate receptor that play diverse roles in neuronal excitability, pre‐synaptic release and post‐synaptic signalling (Evans, Gurung, Henley, Nakamura, & Wilkinson, 2019). Several studies have shown that the KAR subunit GluK2 is SUMOylated at a single C‐terminal lysine, K886, in an agonist‐dependent manner, resulting in endocytosis of GluK2‐containing KARs (Figure 1, for reviews see (Pahl, Tapken, Haering, & Hollmann, 2014; Henley et al., 2018)). Intriguingly, it has also been reported that GluK2 internalization following SUMOylation promotes binding to mixed lineage kinase 3 (MLK3) and activation of the MLK3‐c‐Jun *N*‐terminal kinase 3 (JNK3) pathway, which could contribute to neuronal loss in ischemia (Zhu, Xu, Du, & Hou, 2012).

Another interesting observation is that KAR activation causes endocytosis of GlyRs, which are important inhibitory receptors that have been extensively studied in spinal cord. This effect is calcium and PKC‐dependent. Moreover, the authors show that PKC is a SUMO substrate and that SUMOylation inhibits its activity. Furthermore, KAR‐evoked GlyR endocytosis requires SENP1‐mediated deSUMOylation of PKC, leading to enhanced phosphorylation of GlyR (Sun et al., 2014) (Figure 1).

More recently, the same group also reported that SUMOylation of PKC inhibits the binding of 14–3‐3tau to GluK2 by reducing GluK2 phosphorylation (Li, Wang, Zhu, Zhou, & Li, 2017). 14–3‐3tau is a conserved family of regulatory proteins that bind to a wide range of targets, and binding to GluK2 contributes to the slow decay kinetics of KAR‐mediated excitatory post‐synaptic currents (EPSCs) (Sun et al., 2013). Together, these studies suggest that PKC SUMOylation may be an important modulator of synaptic (and other) proteins by regulating PKC phosphorylation. Indeed, given that SUMOylation of GluK2 is itself promoted by GluK2 phosphorylation by PKC (Chamberlain et al., 2012; Konopacki et al., 2011), and the role of PKC in stabilizing the synaptic residence time of Ubc9 (Loriol et al., 2014), these data suggest a sophisticated regulatory interplay between SUMOylation and PKC.

#### 3.2.5 Dopamine transporter (DAT)

Dopamine transporter (DAT) reuptakes released dopamine into the pre‐synaptic terminal to terminate dopamine neurotransmission. DAT function is influenced by cocaine and amphetamine and its dysfunction has been strongly implicated in neurological conditions including autism spectrum disorders, attention‐deficit hyperactivity disorder (ADHD) and Parkinson's disease (German, Baladi, McFadden, Hanson, & Fleckenstein, 2015). The surface expression of DAT is critical for its function and a recent study has reported that SUMO1‐ylation acts to enhance the total levels of DAT in the plasma membrane (Cartier et al., 2019). Imaging and western blot assays revealed the close association of SUMO1 and DAT, and over‐expression of SUMO1 or Ubc9 enhanced DAT surface expression and stability. Moreover, expression of SUMO1 reduced DAT ubiquitination. Conversely, Ubc9 knock‐down caused a decrease in SUMO‐DAT association and an increase in DAT degradation (Cartier et al., 2019). However, because site directed mutagenesis of lysines that are predicted as SUMOylation sites has not been reported, it has not yet been unequivocally established whether it is covalent SUMO1 conjugation or non‐covalent association with SUMO or a SUMOylated interactor through a SUMO interaction motif (SIM) that regulates DAT surface expression and turnover. Indeed, one of the three predicted SIMs contain a lysine (K35) that is targeted for ubiquitination (Miranda, Dionne, Sorkina, & Sorkin, 2007; Miranda, Wu, Sorkina, Korstjens, & Sorkin, 2005) suggesting that SUMO binding might be sufficient to impair ubiquitin‐mediated degradation. Nonetheless, these data indicate a role for SUMO in the regulation of DAT and, consequently, in dopamine signalling.

## 4 Direct regulation of ion channels by SUMOylation

Given the diversity and number of reports on ion channel subunit SUMOylation it seems likely that this post‐translational modification, through altering ion channel conformation, acting to block the channel pore, or enhancing or inhibiting protein interactions, may represent a common mechanism for their functional regulation (for recent review see (Benson, Iniguez‐Lluhi, & Martens, 2017)).

For example, a recent study using the lateral pyloric neuron of spiny lobsters showed that SUMO conjugation and deconjugation is synergistically regulated to control the opposing hyperpolarization activated current (I_h_) mediated by hyperpolarization‐activated cyclic nucleotide–gated (HCN) channels, and the transient potassium current (I_A_) mediated by K_v_4 channels (Parker, Forster, & Baro, 2019). These data suggest that activity‐dependent SUMOylation can rapidly adjust ionic current densities to homeostatically control the balance of neuronal excitation by altering surface expression and biophysical properties (Parker et al., 2016; Welch, Forster, Atlas, & Baro, 2019), highlighting SUMOylation as a potential ‘key coordinator’ of neuronal excitability.

### 4.1 K^+^ and Na^+^ ion channels

The two‐pore potassium channel, K2P1, which acts to suppress neuronal excitability, was the first identified ion channel to be SUMOylated. In this case, constitutive SUMOylation prevents channel activity (Rajan, Plant, Rabin, Butler, & Goldstein, 2005). Subsequent studies reported that multiple voltage‐dependent potassium (K_v_) channels are also SUMOylated. SUMOylation of K_v_1.5 regulates channel inactivation (Benson et al., 2007), indicating that while SUMOylation entirely inhibits K^+^ conductance through K2P1, the effects of SUMOylation of K_v_1.5 are more nuanced. It has also been reported that K_v_4.2 channels are SUMOylated at two distinct sites (K437 and K579) which increase surface expression and decrease potassium current maximal conductance respectively (Welch et al., 2019). Additionally, SUMOylation decreases K_v_2.1 (Plant, Dowdell, Dementieva, Marks, & Goldstein, 2011), K_V_7.2 (Qi et al., 2014), K_V_7.1 (Xiong et al., 2017) and K_V_11.1 (Steffensen, Andersen, Mutsaers, Mujezinovic, & Schmitt, 2018) currents, highlighting this modification as general regulator of potassium channel function.

**FIGURE 2:**
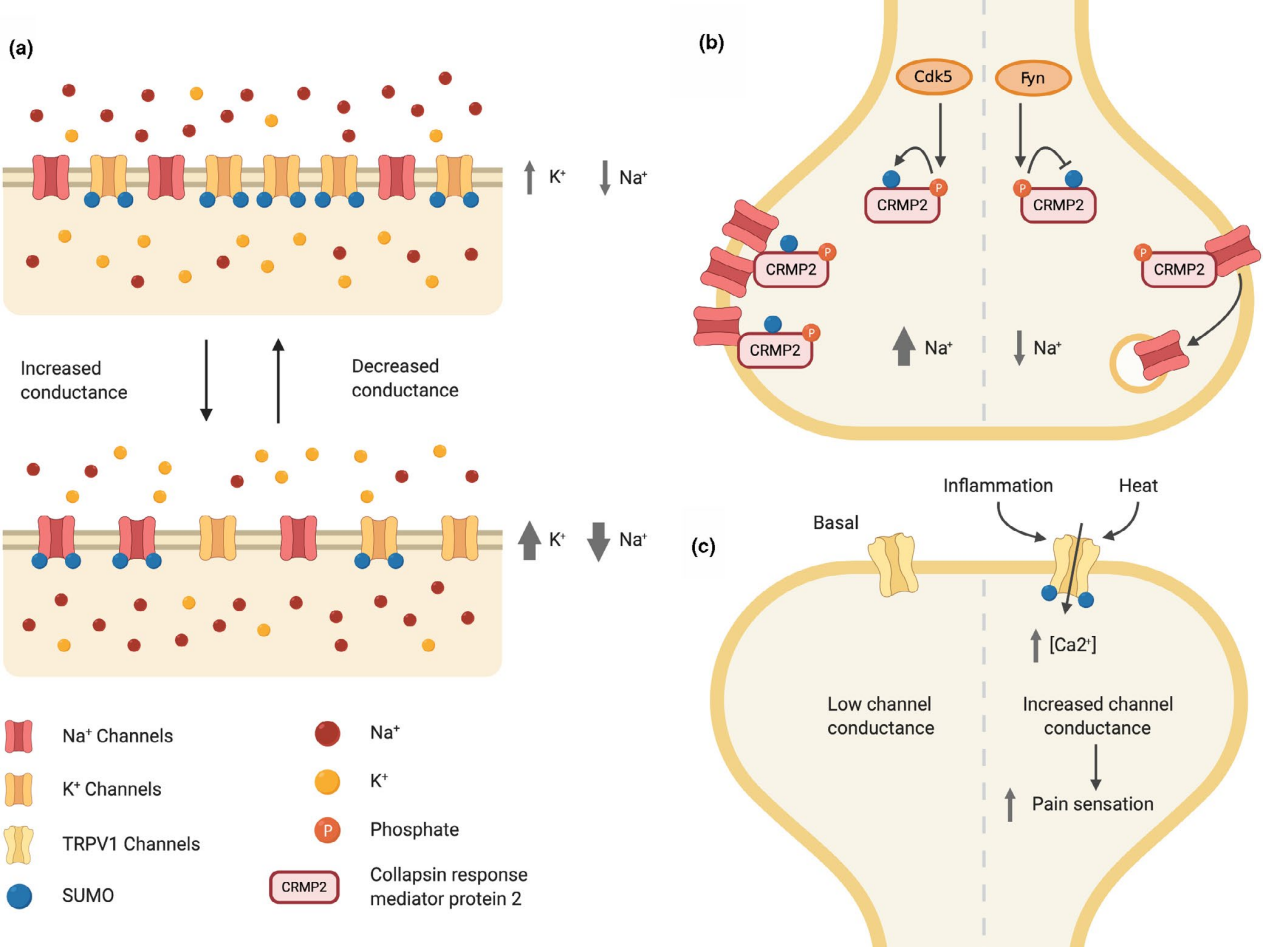
Ion channel SUMOylation. (a) Overall, SUMOylation of potassium channels reduces conductance, whereas SUMOylation of sodium channels increases conductance. (b) Cdk5 phosphorylation enhances CRMP2 SUMOylation, whereas Fyn phosphorylation antagonizes its SUMOylation. SUMOylated CRMP2 increases Na_V_1.7 sodium channel surface expression and current density. (c) Inflammation increases TRPV1 channel SUMOylation, which reduces the threshold of channel activation by heat and increases pain sensation

Conversely, SUMOylation of the voltage‐dependent sodium channel Na_V_1.2 increases Na^+^ currents (Plant, Marks, & Goldstein, 2016). Thus, the current literature indicates that the overarching effect of ion channel SUMOylation is to increase neuronal excitability by suppressing K^+^ channels and enhancing Na^+^ channels (Figure 2). Since SUMOylation of these channels is likely differentially regulated under different stimulation conditions, how SUMOylation coordinates and integrates their activity to control neuronal excitability, analogous to its homeostatic regulation of the I_h_ and I_A_ currents, is an important question for future research.

### 4.2 Ligand‐gated non‐selective cation channel TRPV1

TRPV1 (also known as the capsaicin receptor, vanilloid receptor 1) is abundantly expressed in dorsal root ganglia (DRG) neurons (Andresen, 2019), and is necessary for the development of inflammatory thermal hyperalgesia, a phenomenon whereby inflammation leads to enhanced sensitivity to thermal stimuli. TRPV1 is a SUMO substrate and its SUMOylation at K822 in DRG neurons is enhanced by peripheral inflammation (Wang et al., 2018). SUMOylation at K822 lowers the threshold for TRPV1 channel activation by heat, increasing sensitivity to thermal stimuli at the inflamed site (Figure 2). Conditional knockout mice in which SENP1 was ablated selectively from DRG neurons to prolong protein SUMOylation displayed greater inflammatory thermal hyperalgesia (Wang et al., 2018). Conversely, TRPV1 knockout mice are resistant to inflammatory thermal hyperalgesia. This is reversed by virally mediated expression of wild‐type, but not SUMOylation‐deficient (K822R), TRPV1 in DRG neurons. These data suggest that SUMO1‐ylation of TRPV1 sensitizes the channel to heat activation (Wang et al., 2018), and provides an elegant example of the importance of SUMOylation of an individual target protein in underpinning a complex physiological behaviour.

## 5 Indirect regulation of ion channels by SUMOylation

### 5.1 Collapsin response mediator protein 2 (CRMP2)

Collapsin response mediator protein 2 (CRMP2) is a member of a cytosolic family of proteins that dynamically regulate microtubule stability and is involved in axon–dendrite polarity, dendritic spine development and synaptic plasticity, and its dysfunction is strongly implicated in neuropsychiatric disease (Quach, Honnorat, Kolattukudy, Khanna, & Duchemin, 2015). Moreover, CRMP2 appears to play a pivotal role in the assembly and function of channel complexes including, but not limited to, voltage‐gated ion channel assemblies involved in pain transmission (Chew & Khanna, 2018).

CRMP2 has been identified as a SUMO substrate and, initially, SUMOylation of CRMP2 was shown to decrease calcium influx mediated by Ca_V_2.2 calcium channels (Ju et al., 2013) but has now been most extensively studied for its regulation of Na_V_1.7 sodium channels (for recent review see (Chew & Khanna, 2018)). The importance of CRMP2 in pain transmission is well established and is the subject of concerted efforts to determine if it is a druggable therapeutic target (Chew, Bellampalli, Dustrude, & Khanna, 2019; Chew & Khanna, 2018).

The first study indicating that SUMOylation of CRMP2 regulates sodium channel trafficking and biophysical properties showed that expression of a non‐SUMOylatable CRMP2 (CRMP2‐K374A) reduced Na_V_1.7 current density and surface expression in the catecholamine A differentiated neuroblastoma cell line, and reduced sodium current density in DRG neurons. These effects on Na_V_1.7 current density and surface expression could also be reproduced by promoting deSUMOylation with SENP1 and SENP2 in CRMP2‐expressing cells (Dustrude, Wilson, Ju, Xiao, & Khanna, 2013).

A later study showed that expression of a non‐SUMOylatable point mutant (K374A) of CRMP2 reduces CRMP2 binding to Na_V_1.7 and promotes clathrin‐dependent Na_V_1.7 internalization (Dustrude et al., 2016). They also observed that interplay between phosphorylation and SUMOylation of CRMP2 controls CRMP2 SUMOylation and NaV1.7 trafficking. Cyclin‐dependent kinase 5 (CK5)‐mediated phosphorylation of CRMP2 at S522 enhances its SUMOylation, whereas phosphorylation by Fyn at Y32 antagonizes CRMP2 SUMOylation and promotes Na_V_1.7 internalization, leading to decreased Na_V_1.7 membrane localization, current density and neuronal excitability (Figure 2).

CRMP2 SUMOylation is increased during an in vivo model of neuropathic pain, consistent with it increasing Na_V_1.7 surface expression (Moutal et al., 2018). Experiments expressing either CRMP2 WT or CRMP2 K374A in vivo showed that CRMP2 K374A significantly reversed mechanical allodynia—pain caused by a stimuli that does not normally illicit a painful response—in a spared nerve injury model of neuropathic pain, suggestive of a functional link between CRMP2 SUMOylation and pain (Moutal et al., 2018). Intriguingly, a TAT‐peptide that interrupts SUMOylation of CRMP2 causes similar suppression of Na_V_1.7 currents. Infusion of this peptide reversed persistent hypersensitivity in an in vivo spinal nerve injury model of neuropathic pain (Francois‐Moutal et al., 2018 et al. 2018), providing an exciting proof‐of‐concept for the strategy of using cell‐permeable peptides targeting SUMOylation of individual substrates for therapeutic benefit.

#### 5.1.1 CRMP2 and dendritic spines

It has been reported recently that CRMP2 over‐expression increases the size and frequency of miniature excitatory post‐synaptic currents (mEPSCs), the number of PSD95 puncta and the density of mature, mushroom‐shaped spines in hippocampal neurons (Zhang et al., 2018). Furthermore, preventing phosphorylation of CRMP2 at T514, or SUMOylation at K374, further enhanced the frequency and amplitude of mEPSCs and the number of mature spines. Surprisingly, however, these authors found no evidence of interplay between these two post‐translational modifications, suggesting that they may act via separate pathways to regulate spine maturation (Zhang et al., 2018).

## 6 SUMOylation of other proteins involved in the regulation of synaptic function

### 6.1 Arc

Expression of the cytoskeleton‐associated immediate early gene Arc is rapidly induced by neuronal activity to participate in various forms of synaptic plasticity, including LTP, LTD and homeostatic scaling (Carmichael & Henley, 2018). It plays pivotal roles in regulating AMPAR trafficking (Chowdhury et al., 2006; Shepherd et al., 2006), memory formation (Zhang et al., 2019) and trafficking of the Alzheimer's associated proteins amyloid precursor protein and beta‐site amyloid precursor protein cleaving enzyme 1 (β‐secretase 1) (Wu et al., 2011).

**FIGURE 3:**
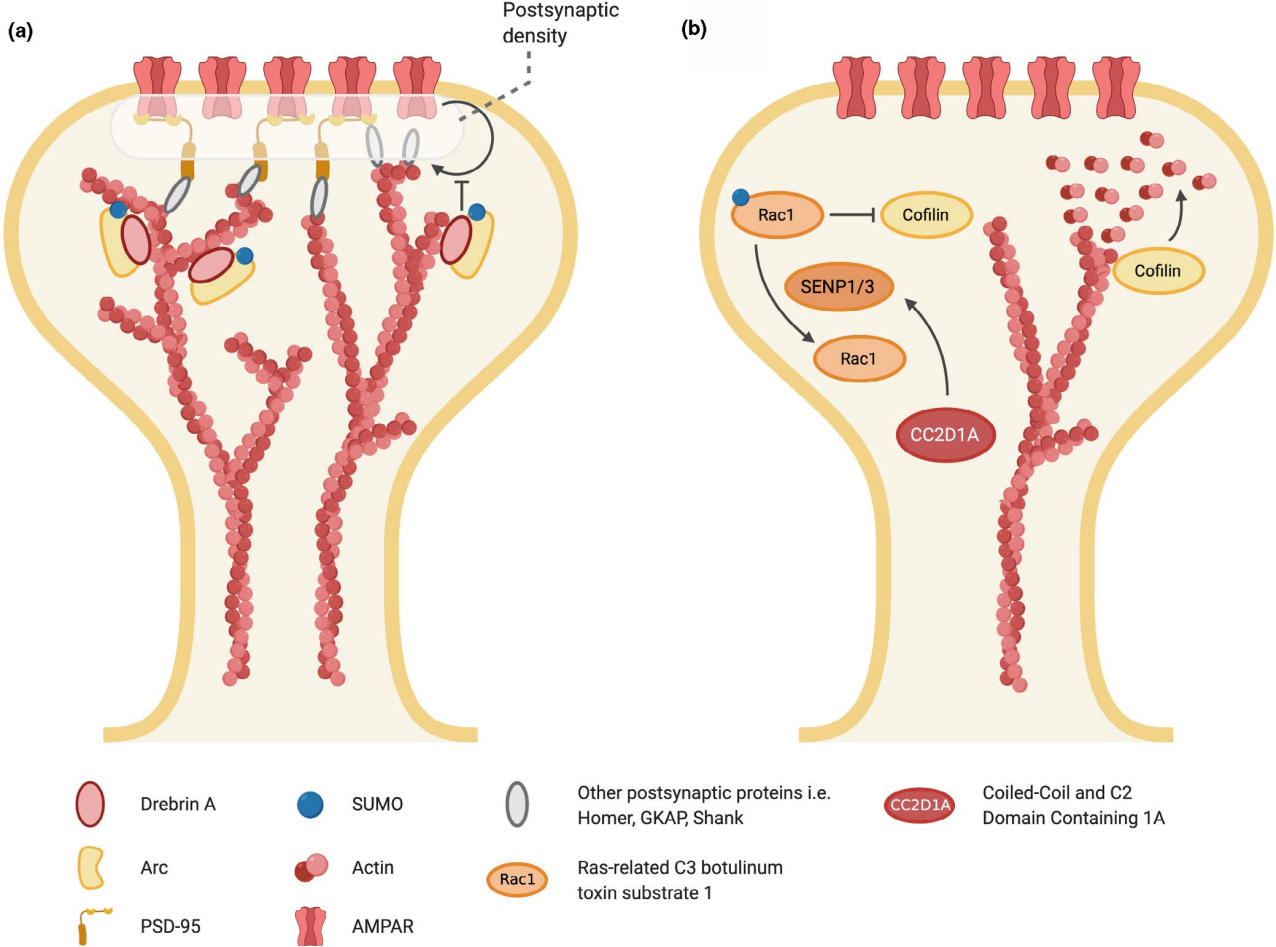
SUMOylation of GTPases during synaptic plasticity. During LTP, SUMOylated Arc associates with drebrin A, an actin‐binding protein. This impairs α‐amino‐3‐hydroxy‐5‐methyl‐4‐isoxazolepropionic acid receptor (AMPAR) internalization to promote increased surface expression. (a) CC2D1A deletion leads to reduced SENP 1/3 levels, which in turn, regulates the SUMOylation of Rac1. SUMOylated Rac1 enhanced Rac1 activity. Rac1 inhibits cofilin activity, which severs F‐actin. SUMOylation of Rac1 enhances activity, thus indirectly promotes actin stabilization by blocking cofilin in dendritic spine formation

Arc is subject to multiple post‐translational modifications including phosphorylation, ubiquitination and SUMOylation (Carmichael & Henley, 2018; Mabb & Ehlers, 2018). In particular, Arc SUMOylation has been proposed as an important determinant of protein‐protein interactions and function in homeostatic synaptic scaling (Craig & Henley, 2012) and LTP induction in vivo (Nair et al., 2017). Arc synthesized during the maintenance phase of LTP induced in vivo undergoes enhanced SUMO‐1‐ylation, which is prevented if LTP maintenance is inhibited (Nair et al., 2017). SUMOylated Arc was detected in synaptosomal and cytoskeletal fractions and complexes with the F‐actin‐binding protein drebrin A, a regulator of dendritic spine cytoskeletal dynamics (Shirao et al., 2017), raising the possibility that newly synthesized Arc is SUMOylated and targeted for actin cytoskeletal regulation during a LTP (Nair et al., 2017); Figure 3.

#### 6.1.1 FMRP

Group I mGluRs (mGluR1 and mGluR5) are mainly post‐synaptic and activate a range of downstream pathways. Activation of Group I mGluRs induces a form of LTD and their dysfunction has been strongly implicated in disease (Ribeiro, Paquet, Cregan, & Ferguson, 2010). Importantly, in addition to regulating the synaptic retention of Ubc9 and SENP1 discussed previously (Loriol et al., 2014; Schorova et al., 2019), mGluR5 activation increases SUMOylation of fragile X mental retardation protein (FMRP), which leads to the dissociation of FMRP from dendritic mRNA granules to promote spine maturation (Khayachi et al., 2018) (Figure 1). This is important because abnormal dendritic spine maturation leads to Fragile X syndrome, a major cause of intellectual disability and the most well characterized monogenic cause of autism. Thus, through control of FMRP, SUMOylation potentially plays key roles in synapse maturation and neuronal network formation (Khayachi et al., 2018). Indeed, a role for SUMOylation in controlling dendritic spine number has also been shown in mice over‐expressing SUMO1 (Matsuzaki et al., 2015). Cortical and hippocampal neurons from SUMO1 over‐expressing mice exhibited reduced spine density, and mice displayed defective fear conditioning responses (Matsuzaki et al., 2015). While this may seem inconsistent with the requirement for FMRP SUMOylation to promote spine maturation, it is important to note that SUMO1 over‐expression will lead to enhanced SUMOylation of many substrates, some of which may have effects on spine number. Thus, while SUMOylation of FMRP promotes spine maturation, the net effect of globally enhancing SUMO1‐ylation is decreased spine number. However, the identity of these other SUMOylated spine regulators remains to be determined.

### 6.2 α‐SYNUCLEIN

The pre‐synaptically localized protein α‐synuclein has been proposed to play roles in regulating the size of the synaptic vesicle pool and assembly of the neurotransmitter release machinery (Burre et al., 2010). α‐Synuclein is also the focus of concerted research because of its association with both sporadic and familial forms of Parkinson's Disease (PD). α‐Synuclein‐containing aggregates known as Lewy Bodies are a hallmark of PD, and, so far, 6 mutations in α‐synuclein have been associated with familial PD (Dehay & Fernagut, 2016). α‐Synuclein was first identified as a SUMO substrate by Dorval and Fraser (Dorval & Fraser, 2006), and subsequent work demonstrated that 11 of the 15 lysines in α‐synuclein can be SUMOylated (Kim et al., 2011; Krumova et al., 2011; Oh, Kim, Mouradian, & Chung, 2011; Rousseaux, de Haro, & Lasagna‐Reeves, 2016; Rousseaux et al., 2018). SUMOylation has been variously proposed to regulate α‐synuclein localization, turnover, aggregation and toxicity (Kim et al., 2011; Krumova et al., 2011; Oh et al., 2011; Rousseaux et al., 2016, 2018), suggesting that many aspects of α‐synuclein behaviour are under the control of SUMOylation, potentially depending on the specific lysines modified, the cellular context, and experimental system used. Most recently, interplay between SUMOylation and ubiquitination has been shown to control α‐synuclein turnover (Rott et al., 2017). Rott *et al* identified PIAS2 as an E3 ligase that promotes α‐synuclein SUMOylation. Expression of PIAS2 in HEK293 cells also reduced mono‐ubiquitination of α‐synuclein and enhanced its stability, suggesting SUMOylation negatively regulates ubiquitin‐mediated α‐synuclein degradation. Interestingly, three PD‐associated α‐synuclein mutants displayed enhanced SUMOylation, which was promoted by PIAS2, leading to the formation of α‐synuclein aggregates. Moreover, levels of PIAS2, and SUMOylated α‐synuclein, were increased in the cerebral cortex of PD patients who progressed to dementia (Rott et al., 2017). Together, these data suggest that enhanced PIAS2 levels in PD may contribute to disease progression through enhancing the SUMOylation and aggregation of α‐synuclein. Moreover, it remains possible that SUMOylation also regulates the proposed functions of α‐synuclein in pre‐synaptic neurotransmitter release under non‐pathological conditions, although this has not yet been investigated.

### 6.3 Neuronal nitric oxide synthase

Neuronal nitric oxide synthase (nNOS) and nitric oxide (NO) signalling can mediate long‐lasting synaptic plasticity. A very recent paper has shown that NMDAR stimulation promotes SUMO1‐ylation of nNOS at K725 and K739. nNOS SUMOylation enhances its phosphorylation at S1412, which increases NO production (Du et al., 2020). The authors reported that nNOS SUMOylation is required for NMDAR‐evoked activation of ERK and hippocampal LTP induction, and that blocking activity‐induced nNOS SUMOylation suppressed LTP‐related expression of Arc and brain‐derived neurotrophic factor (Du et al., 2020).

### 6.4 Small GTPases

Small GTPases function as ‘molecular switches’ to coordinate a multitude of cellular signalling pathways (Hall, 1990). In neurons, several small GTPases play crucial roles in controlling neuronal morphology, synapse formation (Gonzalez‐Billault et al., 2012; Woolfrey & Srivastava, 2016), and regulating the synaptic actin cytoskeleton and post‐synaptic AMPAR trafficking during synaptic plasticity (Hotulainen & Hoogenraad, 2010; Kruijssen & Wierenga, 2019; Patterson, Szatmari, & Yasuda, 2010).

#### 6.4.1 Rac1

Rac1, and the related GTPases RhoA and Cdc42, have been identified as causal signals for the initiation of changes in spine structure and structural LTP (Hedrick et al., 2016). The stabilization of the f‐actin cytoskeleton is pivotal to structural remodelling of dendritic spines late phase LTP and this is regulated in part by preventing the disassembly of actin filaments by cofilin A (Bosch et al., 2014; Hlushchenko, Koskinen, & Hotulainen, 2016). Rac 1 inhibits actin depolymerization by cofilin indicating that Rac 1‐cofilin signalling plays a key role in spine morphology (Pyronneau et al., 2017) (Figure 3).

Rac1 was the first member of the Ras family GTPases shown to be SUMOylated (Castillo‐Lluva et al., 2010). SUMOylation enhances Rac1 activation (Castillo‐Lluva et al., 2010), and subsequent work has demonstrated a link between Rac1 SUMOylation, LTP and performance in object location memory tests in vivo (Yang, Yu, Wen, Ling, & Hsu, 2019). Rac1 SUMOylation is at least partly regulated by expression levels of SENP1 and SENP3, which are in turn regulated by the coiled‐coil and C2 domain‐containing 1A (CC2D1A) protein (Yang et al., 2019). CC2D1A has multiple roles including acting as a transcriptional repressor (Szewczyk et al., 2010) and as a scaffold/regulator of protein kinases (Al‐Tawashi, Jung, Liu, Su, & Qin, 2012; Nakamura, Naito, Tsuruo, & Fujita, 2008), and mutations in CC2D1A cause autosomal recessive non‐syndromic intellectual disability (Al‐Tawashi et al., 2012; Nakamura et al., 2008). Conditional knock‐out of CC2D1A in excitatory neurons results in impaired hippocampal LTP maintenance and reduced performance in object location memory tests (Yang et al., 2019). Intriguingly, loss of CC2D1A reduces SENP1/3 levels, which enhances SUMOylation and activation of Rac1. Since LTP and object location memory can be rescued in these mice by pharmacological inhibition of Rac1, the authors propose that CC2D1A controls synaptic plasticity and memory by regulating SENP levels and SUMOylation of Rac1 (Yang et al., 2019) (Figure 3).

#### 6.4.2 Ras

Ras activation drives the delivery and surface expression of AMPARs during LTP (Zhu, Qin, Zhao, Van Aelst, & Malinow, 2002). All three isoforms of Ras (H‐Ras, K‐Ras and N‐Ras) can be modified by SUMO2/3 at a single lysine, K42 (Choi, Chen, Philips, & Dai, 2018). SUMOylation of K‐Ras is enhanced by the E3 ligase PIASγ (Choi, Chen, et al., 2018), and mutation of the SUMO acceptor site leads to a reduction in activation of the downstream targets of Ras, c‐Raf, MAPK/ERK kinase 2 and ERK (Choi, Philips, Chen, Lu, & Dai, 2018), suggesting SUMOylation of Ras promotes Ras signalling. While the roles of Ras SUMOylation have not yet been directly investigated in neurons, these wider findings offer the intriguing possibility that Ras SUMOylation may play a role in LTP through regulation of AMPAR trafficking.

#### 6.4.3 Rhes‐mediated SUMOylation of Huntingtin

Ras homolog enriched in the striatum (Rhes) (Falk et al., 1999) is a small GTPase that also functions as a SUMO E3 ligase to enhance SUMOylation of poly‐Q expanded mutant Huntingtin (mHTT), the protein that is the cause of the lethal neurodegenerative disorder Huntington's disease (HD) (Harrison & Lahoste, 2013). Although HD is characterized by aggregates of poly‐Q expanded mHtt, evidence suggests that the soluble form represents the toxic species (Arrasate & Finkbeiner, 2012; Harrison & Lahoste, 2013). SUMOylation reduces mHtt aggregation by enhancing its solubility (Steffan et al., 2004; Subramaniam, Sixt, Barrow, & Snyder, 2009). Interestingly, Rhes only promotes SUMOylation of mHtt, not wild‐type Htt, thereby reducing aggregation of mHtt and increasing the amounts of the toxic soluble form (Subramaniam et al., 2009, 2010; Subramaniam & Snyder, 2011). Thus, the high expression of Rhes may underlie the sensitivity of the striatum to neurodegeneration in HD. Based on this hypothesis, bioinformatic analyses and molecular modelling of the interaction domains between Rhes, mHtt and Ubc9 are currently being used to design inhibitory peptides to combat HD (Carbo et al., 2019).

#### 6.4.4 Rab17

Another small GTPase involved in polarized trafficking, dendritic morphogenesis and post‐synaptic development in hippocampal neurons is Rab17 (Mori, Fukuda, & Henley, 2014; Mori, Matsui, Furutani, Yoshihara, & Fukuda, 2012). In cultured neurons, Rab17 co‐localizes with Syntaxin‐4, a post‐synaptic SNARE protein that has been implicated in the exocytosis of AMPARs in dendritic spines (Kennedy, Davison, Robinson, & Ehlers, 2010; Mori et al., 2014). Rab17 knockdown significantly reduced surface expression of the KAR subunit GluK2, but did not affect the AMPAR subunit GluA1, and causes Syntaxin‐4 redistribution away from dendrites and into axons in developing hippocampal neurons (Mori et al., 2014). Moreover, over‐expression of constitutively active Rab17 promoted dendritic surface expression of GluK2 by enhancing Syntaxin‐4 translocation to dendrites. These data suggest that Rab17 mediates the dendritic trafficking of Syntaxin‐4 to selectively regulate dendritic surface insertion of GluK2‐containing KARs in rat hippocampal neurons (Mori et al., 2014). Intriguingly, in analogous studies, mono‐SUMOylation of Rab17 has also been shown to selectively promote its interaction with Syntaxin‐2 in polarized liver cells, although these authors did not detect an effect of Rab17 SUMOylation on interaction with Syntaxin‐4 in these cells. Expression of non‐SUMOylatable Rab17(K68R) redistributes Syntaxin‐2 from the apical membrane to subapical puncta. Furthermore, preventing Rab17 SUMOylation reduced apical vesicle docking (Striz & Tuma, 2016) which is required for transcytotic vesicle delivery (Striz, Stephan, Lopez‐Coral, & Tuma, 2018). To our knowledge, the role(s) of Rab17 SUMOylation in neurons has not been explored, but these findings suggest SUMOylation may play a role in the polarized insertion of neurotransmitter proteins into the neuronal membrane.

### 6.5 Regulation of local dendritic protein synthesis; CPEB3

LTP is maintained and memory is encoded by the local synthesis of essential synaptic proteins (Kandel, Dudai, & Mayford, 2014). This local protein synthesis is regulated by cytoplasmic polyadenylation element‐binding protein 3 (CPEB3), which exists in either a soluble inactive form, or an aggregated, insoluble, active form (Fioriti et al., 2015). Under basal conditions CPEB3 is SUMOylated by SUMO2/3 and acts as a local translational repressor (Drisaldi et al., 2015). However, following stimulation of hippocampal neurons with glycine to chemically induce LTP, CPEB3 is rapidly deSUMOylated, which allows it to oligomerize, aggregate and regulate translation of its target proteins Intriguingly, SUMO2 mRNA is also a target of CPEB3, consistent with a negative regulatory feedback mechanism in which CPEB3 drives expression of SUMO2, presumably limiting the extent and duration of CPEB3 activity (Drisaldi et al., 2015). A more recent study has examined in detail how SUMOylation of CPEB3 allows it to switch from a repressor to an activator of local translation (Ford, Ling, Kandel, & Fioriti, 2019). Ford *et al*. demonstrated that under basal conditions, SUMOylated CPEB3 is localized to membraneless cytoplasmic processing bodies (P‐bodies) that contain translationally repressed mRNAs. Following stimulation, CPEB3 is deSUMOylated and recruited into polysomes to actively translate target mRNAs (Ford et al., 2019). Together, these data show that dendritic SUMOylation acts as a negative regulator of CPEB3 aggregation, and that activity‐dependent deSUMOylation of CPEB3 is required for the translation of its target mRNAs during synaptic plasticity.

## 7 CONCLUSIONS AND OUTLOOK

In addition to the important roles of nuclear SUMOylation in neurons, there is substantial, diverse and compelling evidence that SUMOylation of proteins outside the nucleus regulates synaptic activity, plasticity and neuronal excitability. The direct SUMOylation of proteins at, or close to, the synapse has been questioned (Daniel et al., 2017; Tirard et al., 2012) [but see (Henley et al., 2018; Wilkinson et al., 2017)]. However, as evidenced by the examples cited here, we believe that the essential roles of SUMOylation of synaptic and synapse‐associated proteins on neurotransmission, synaptic plasticity and memory is well established.

Nonetheless, there is still a great deal we do not know about the roles of SUMOylation in the brain. Recent advances in the large‐scale identification of SUMO substrates by mass spectrometry (Hendriks & Vertegaal, 2016) will undoubtedly advance the field. We anticipate future studies will provide further evidence for the breadth of neuronal proteins, both nuclear and extranuclear, that are modified by SUMO.

Moreover, while an expanding repertoire of synaptic and synapse‐associated proteins has, and continues to be, identified as SUMO substrates, the regulatory mechanisms involved remain elusive. For example, how are the restricted set of enzymes that conjugate or remove SUMO from target proteins controlled to orchestrate the differential modification of these substrates under basal and activity‐dependent conditions? For many substrates, SUMOylation is regulated by interplay with other post‐translational modifications, including phosphorylation and ubiquitination (Wilkinson & Henley, 2010). Given the well‐established role of these post‐translational modifications in plasticity (Widagdo, Guntupalli, Jang, & Anggono, 2017; Woolfrey & Dell'Acqua, 2015), it seems highly likely that their interaction with SUMOylation acts to tune synaptic responsiveness. Indeed, whether synapses constitute a site of ‘group modification’, whereby a multitude of SUMO substrates are dynamically modified in a restricted cellular location, as has been demonstrated at sites of DNA strand breaks in non‐neuronal cells (Psakhye & Jentsch, 2012), represents an exciting possibility for future investigation. Finally, given the number of neuronal disease‐associated proteins modified by SUMO, the role of global SUMOylation in cell stress responses, and reports of perturbed SUMOylation in a number of disease states (Henley et al., 2014), targeting the SUMOylation pathway for therapeutic benefit in disorders of the nervous system represents an attractive possibility that is being actively investigated in a number of contexts. Addressing these questions will undoubtedly advance understanding of the mechanisms and multiple roles of neuronal protein SUMOylation and will provide exciting and important new insights into neuronal function and dysfunction.

8

**Table 1:** Summary of SUMO substrates discussed in this review. The table summarizes the sites of modification and SUMO paralogues that target the proteins discussed. Where known, E3s and deSUMOylating enzymes that have activity against the target protein are indicated. Question marks (?) indicate instances where truncated or isolated catalytic domains of SENPs have been used to deSUMOylate the target protein, which may not reflect SENP substrate specificity in vivo

	SUMO site	SUMO paralogues	E3s	SENPs	Role of SUMOylation	References
Synaptic neurotransmitter receptors and transporters						
mGluR7	K889	SUMO1/3	Binds PIAS1 and PIAS3L	SENP1	Enhances surface stability of mGluR7	Choi et al., (2016), Dutting et al., (2011), Tang et al., (2005)
mGluR8b	K882	SUMO1	PIAS1; Binds PIAS3L	Unknown	Unknown	Dutting et al., (2011), Tang et al., (2005)
M1 mAChR	K327	SUMO1	Unknown	Unknown	Promotes ligand‐binding affinity and signal transduction	Xu et al., (2019)
GluK2	K886	SUMO1	Binds PIAS3	SENP1?	Promotes agonist‐induced KAR internalization	Martin et al., (2007)
DAT	Unknown	SUMO1	Unknown	Unknown	Promotes DAT stability and surface expression	Cartier et al., (2019)
Ion channels and associated proteins						
K2P1	K274	SUMO1	Unknown	SENP1	SUMOylation silences the channel	Rajan et al., (2005)
K_v_1.5	K221, K536	SUMO1−3	Unknown	SENP2?	Alters the voltage dependence of steady‐state inactivation	Benson et al., (2007)
K_v_2.1	K470	SUMO1	Unknown	SENP1	Inhibits channel currents by promoting desensitization; positive‐shifts the half maximal activation voltage	Dai, Kolic, Marchi, Sipione, and Macdonald, (2009), Plant et al., (2011)
K_v_4.2	K437, K579	SUMO2/3	Unknown	Unknown	Increased surface expression and decreased maximal conductance	Welch et al., (2019)
K_v_7.1	K424	SUMO1/2	Unknown	SENP2	Causes a positive shift in the half maximal activation voltage	Xiong et al., (2017)
K_v_7.2	Unknown	SUMO1−3	Unknown	SENP2	Reduces channel currents	Qi et al., (2014)
K_v_11.1	K21, K93, K116	SUMO1/2	Unknown	Unknown	Reduces channel currents	Steffensen et al., (2018)
Na_v_1.2	K38	SUMO1	Unknown	SENP1?	Increases channel currents	Plant et al. (2016)
TRPV1	K822	SUMO1	Unknown	SENP1	Enhances channel sensitivity to activation by heat	Wang et al., (2018)
CRMP2	K374	SUMO1−3	Unknown	SENP1, SENP2	Decreases calcium flux by Ca_v_2.2 channels; Promotes Na_v_1.7 surface expression	Dustrude et al., 2013; Ju et al., (2013)
Synaptic and synapse‐associated proteins						
Synapsin 1a	K687	SUMO1	Unknown	SENP1?	Promotes binding to synaptic vesicles (SVs), required for normal levels of SV exocytosis	Tang et al., (2015)
FMRP	K88, 130, 614	SUMO1	Unknown	Unknown	Promotes dissociation of FMRP from RNA granules to promote spine formation	Khayachi et al., (2018)
Arc	K110, K268	SUMO1−3	Unknown	SENP1?	Required for homeostatic upscaling; Promotes interaction with drebrin A during LTP	Craig et al., (2012), Nair et al., (2017)
α‐synuclein	Predominantly K96, K109	SUMO1−3	PIAS2, PC2, TRIM28	Unknown	Effects reported on synuclein localization, stability, aggregation and toxicity	Dorval and Fraser, (2006); Oh et al., (2011); Rousseaux et al. (2018); Rott et al., (2017)
NOS	K725, K739	SUMO1	PIAS3	Unknown	Required for LTP induction and expression of Arc and BDNF	Du et al., (2020)
Small GTPases						
*Rac1*	K183, K184, K186, K188	SUMO1	PIAS3	SENP1 and 3 proposed	Promotes Rac1 activity and downstream signalling	Castillo‐Lluva et al., (2010), Yang et al., (2019)
*Ras*	K42	Predominantly SUMO3	PIASγ	SENP1, SENP2	Required for full activation of downstream signalling pathways	Choi, Chen, et al. (2018))
*Rab17*	K68	SUMO1−3	Unknown	SENP1?	Promotes interaction with Syntaxin−2 and reduces apical vesicle docking	Striz and Tuma, (2016)
Control of local translation						
CPEB3	Unknown	SUMO2/3	Unknown	Unknown	Fusion of SUMO2 to CPEB3 prevents CPEB3 aggregation, repressing local translation	Drisaldi et al., (2015)
Others						
mHTT	K6, K9, K15, K91	SUMO1, SUMO2	Rhes, PIAS1	Unknown	Promotes mHTT toxicity by enhancing its solubility	O'Rourke et al., (2013), Steffan et al., (2004), Subramaniam et al., (2009)
